# The need for mandatory autopsy teaching in Forensic Medicine for medical students

**DOI:** 10.4322/acr.2024.509

**Published:** 2024-08-06

**Authors:** Nurul Kharmila Abdullah, Nur Arina Ahmad, Shalinawati Binti Ramli, Nadiawati Abdul Razak

**Affiliations:** 1 Universiti Sains Islam Malaysia, Forensic Medicine Unit, Nilai, Negeri Sembilan, Malaysia; 2 National Defence University of Malaysia (NDUM), Faculty of Medicine and Defence Health, Forensic Medicine Unit, Sungai Besi, Kuala Lumpur, Malaysia

**Keywords:** autopsy, education, forensic medicine, undergraduate, teaching

## Abstract

The effectiveness of the autopsy as an educational tool in forensic medicine courses has been widely acknowledged, and medical students were expected to attend regularly. Nevertheless, the use of autopsies for teaching has dramatically declined in recent years and worldwide despite their high-value benefits. This study aims to understand the importance and relevance of attending autopsies during forensic teaching sessions and identify any challenges that may impede attendance. A self-administered online questionnaire that assesses the knowledge, attitudes, and practices related to autopsies attendance was distributed to fourth-year medical students at the National Defence University of Malaysia and Universiti Sains Islam Malaysia. A total of 99 respondents were involved in this study. Our findings indicate that most respondents (over 85%) demonstrated good knowledge of forensic medicine. Pearson's statistical test revealed a significant correlation between the knowledge and students' attitudes toward autopsy. This study demonstrates the need to strategically integrate autopsy attendance into medical curricula to encourage constructive attitudes and practices among medical students. Students gain the most benefits from frequently attending autopsies. Passionate educators can conduct preparatory sessions to set expectations and address concerns, encourage students to process their experiences, and reinforce learning outcomes in the mortuary setting. Mandatory autopsy teaching should be integrated into the curriculum to ensure medical students have the necessary skills and knowledge to become competent doctors.

## INTRODUCTION

Forensic medicine, or legal medicine, is mandatory for undergraduate medical students in Malaysia. The goal of the forensic medicine curriculum for medical undergraduates is to equip students with the necessary knowledge and skills to fulfill medicolegal responsibilities in medicine. Autopsies have long been a cornerstone of medical education, providing invaluable insights into the underlying causes of disease and the effects of therapeutic interventions. Autopsy, or post-mortem examination, remains one of the most informative tools for medical education and clinical practice. It gives medical students a direct view of the disease's physiological manifestations, offering a real-world complement to theoretical knowledge and contributing significantly to their comprehensive medical education. Autopsies are generally classified into two main types: (i) medicolegal or forensic autopsies, which are ordered by legal regulations and carried out to establish the cause and circumstances of death and related issues; (ii) hospital or clinical autopsies, which are optional and performed to gain deeper insights into an uncommon illness or its pathogenesis. These procedures provide explanations that may aid both the family and the medical practitioner and assist the family in coping with their grief.^1^

Students must attend autopsy sessions at the hospital, where they are to observe the autopsy examination and procedures. Attendees observe and engage in the autopsy process under supervision, gaining skills in proper technique, documentation, and sample collection. This practical immersion enhances their comprehension of anatomy, pathology, and injuries and their ability to correlate between clinical and pathology knowledge.^2^

Despite its recognized value, autopsy attendance has faced challenges, including declining autopsy teaching rates pointed out by scarce resources and practitioners,^3^ logistical issues, and sometimes a lack of enthusiasm among students or faculties. Even where attendance is encouraged, the level of active participation by students in assisting or attending autopsies often needs to be revised.^3^ Forensic medicine has been taught in medical schools since ancient times and continues to maintain its significance. However, there has been an imminent decline in teaching undergraduates worldwide.^3,4^ This alarming trend arises from a decrease in autopsy rates for patients dying in hospitals, which has dropped steeply over the past 40 years in New Zealand, the United Kingdom, and the United States,^5^ the availability of alternative teaching methodologies, and the stringent enforcement of attendance regulations by law in certain jurisdictions.^5,6^ In New Zealand, medical students are forbidden to be present during the autopsy.^6-8^

In Malaysia, the curriculum for forensic medicine is covered during the fourth year of medical education, which consists of a brief posting that lasts for two weeks. The subjects included in the curriculum cover an array of topics such as death certification, the examination of wounds and injuries, the differentiation between natural, traumatic, and accidental deaths—including asphyxia deaths and those caused by firearms—along with maternal and child mortality, and toxicological studies.

This article explores Malaysian medical students' understanding of the importance and relevance of attending autopsies during their forensic teaching sessions. Furthermore, the study aims to investigate medical students' attitudes towards participating in autopsies and identify any barriers or challenges they may face in attending them.

## METHODS

The research involved 138 participants among 53 National Defence University of Malaysia (NDUM) students and 85 students at Universiti Sains Islam Malaysia (USIM) who completed the forensic medicine course during the 2022/2023 academic year. A structured, self-administered online questionnaire containing ten items in each domain relating to forensic medicine to assess the knowledge, attitudes, and practices related to attendance at autopsies in forensic medicine was constructed. The researchers developed the questionnaire, which underwent content validity verification by experts who evaluated its relevance before distribution to the students on the final day of the course. Individuals who declined to participate or were absent were excluded from the study. All participants provided informed consent before responding. The data were analyzed using SPSS, version 29 (SPSS, Chicago, IL). Frequencies and percentages were used to describe the variables. To investigate the correlation between knowledge, attitude, and practice was determined by Pearson’s test at 95% confidence intervals, it was taken significantly when found Sig. (2-tailed) value <0.05.

## RESULTS

Of the 138 fourth-year invited medical students, 99 participated in the survey, including 61 from USIM and 38 from NDUM. Of these respondents, 65.7% were female—44 from USIM and 21 from NDUM—and 34.3% were male, with 17 from each university. Each student had participated in at least one autopsy session throughout the course.

Table 1 displays the students' understanding of essential forensic medicine principles. Knowledge within the scope of medicolegal, ethics, and forensic toxicology is the component of knowledge most mastered by respondents. K1 and K9 resulted in a perfect score. K7 had the most wrong answers (13 of the 99 students), which assessed the students' knowledge of a specialized technique for bloodless neck dissection in autopsies. In addition, knowledge of the role of autopsies in confirming the deceased's identity (K5) and the necessity to obtain an inquest report for a medicolegal autopsy (K10) is the component of knowledge that students least master. One female student incorrectly answered all three of the aforementioned questions.

**Table 1:** Number and percentage of answers for questions regarding knowledge

	Survey items	Correct answer	Wrong answer
		n (%)	n (%)
K1.	The autopsy can establish the cause of death.	99 (100)	0
K2.	The autopsy is mandatory in all unnatural deaths and sudden deaths outside the hospital.	93 (93.9)	6 (6.1)
K3.	There are two types of autopsies which are medicolegal autopsy and clinical autopsy.	95 (96)	4 (4)
K4.	A clinical autopsy is performed to document the extent of a known disease.	91 (91.9)	8 (8.1)
K5.	The autopsy helps establish the identity of the deceased.	88 (88.9)	11 (11.1)
K6.	Internal examination is mandatory in medicolegal autopsy.	93 (93.9)	6 (6.1)
K7.	The bloodless neck dissection (Prinsloo and Gordon) technique is used in hanging cases.	86 (86.9)	13 (13.1)
K8.	Toxicological analysis is important in road-traffic crash death.	94 (94.9)	5 (5.1)
K9.	Autopsy findings are used as evidence in a court of law.	99 (100)	0
K10.	Before conducting the medicolegal autopsy, the following is necessary: Inquest report (Police 61) from investigating officer	88 (88.9)	11(11.1)

Table 2 shows the attitude of students during the autopsy session. The overall attitude pattern was positive among most students from both universities. 88.9% of students disagree that they are not interested in attending the autopsy because forensic medicine is a minor subject (A9). Most students (90.9%) also disagree that the autopsy demonstration should be conducted virtually via videography teaching instead of physical attendance (A10).

**Table 2:** Analysis of the distribution of respondents’ answers regarding attitudes toward autopsy

			Options									
	Survey items		Strongly disagree		Disagree		Neutral		Agree		Strongly agree	
			N (%)									
A1.	I was comfortable attending the autopsy.	1 (1)		1 (1)		17 (17.2)		38 (38.4)		42 (42.4)	
A2.	I felt that forensic medicine is an interesting and exciting field.	0		4 (4)		15 (15.2)		30 (30.3)		50 (50.5)	
A3.	I was well-prepared mentally and spiritually before observing the autopsy.	0		0		16 (16.2)		39 (39.4)		44 (44.4)	
A4.	I was scared to attend the autopsy because it caused nightmares.	46 (46.5)		32 (32.3)		11 (11.1)		8 (8.1)		2 (2)	
A5.	I do not like the sight and smell of dead bodies.	7 (7.1)		14 (14.1)		48 (48.5)		20 (20.2)		10 (10.1)	
A6.	I cannot stand the long hours of standing during the autopsy.	19 (19.2)		30 (30.3)		30 (30.3)		17 (17.2)		3 (3)	
A7.	I felt that the mortuary was a spooky and haunted place.	32 (32.3)		28 (28.3)		26 (26.3)		11 (11.1)		2 (2)	
A8.	Autopsy provides an opportunity for students to see and touch real diseased organs.	0		1 (1)		4 (4)		17 (17.2)		77 (77.8)	
A9.	I am not interested in attending the autopsy because forensic medicine is a minor subject.	71 (71.7)		17 (17.2)		8 (8.1)		2 (2)		1 (1)	
A10.	The autopsy demonstration should be conducted virtually via videography teaching instead of physical attendance.	80 (80.8)		10 (10.1)		6 (6.1)		2 (2)		1 (1)	

Table 3 shows students’ practice towards autopsy teaching. Most students agree that autopsy experience helps them correlate knowledge with other clinical subjects (P4). None of the students found excuses to skip the autopsy sessions (P5), and 80.8% of the students never left the autopsy session in the middle of the procedure due to the foul smell of the dead body (P3).

**Table 3:** Analysis of the distribution of respondents’ answers regarding practice towards autopsy teaching

		Options					
	Survey items		Never	Rarely	Sometimes	Often	Always
			N (%)				
P1.	I attended extra autopsy sessions.		12 (12.1)	8 (8.1)	30 (30.3)	18 (18.2)	31 (31.3)
P2.	I volunteered to assist the doctor during the autopsy.		9 (9.1)	11 (11.1)	30 (30.3)	24 (24.2)	25 (25.3)
P3.	I left the autopsy session in the middle of the procedure due to the foul smell of the dead body.		80 (80.8)	14 (14.1)	3 (3)	1(1)	1 (1)
P4.	I correlate autopsy experience and knowledge with other clinical subjects.		0	0	21 (21.2)	43 (43.4)	35 (35.4)
P5.	I find excuses to skip the autopsy sessions.		80 (80.8)	15 (15.2)	4 (4)	0	0
P6.	I asked a lot of questions to the doctor during the autopsy session		2 (2)	4 (4)	47 (47.5)	34 (34.3)	12 (12.1)
P7.	I joined the doctor during the explanation of autopsy findings to the grieving family.		44 (44.4)	15 (15.2)	19 (19.2)	12 (12.1)	9 (9.1)
P8.	I recited the prayer in order to respect the deceased		7 (7.1)	10 (10.1)	21 (21.2)	34 (34.3)	27 (27.3)
P9.	I reflected on life after death after attending the autopsy		2 (2)	4 (4)	9 (9.1)	26 (26.3)	58 (58.6)
P10.	I observed the medical attendant/PPK stitching the body after the autopsy		2 (2)	2 (2)	3 (3)	20 (20.2)	72 (72.7)

Pearson's statistical test revealed a significant correlation between knowledge and students' attitudes toward autopsy, as shown in Table 4.

**Table 4:** Pearson correlation

		Attitude	Practice	Knowledge
Attitude	Pearson	1	.109	-.240*
	Sig. (2-tailed)		.285	.016*
	N	99	99	99
				
Practice	Pearson	.109	1	-.050
	Sig. (2-tailed)	.285		.623
	N	99	99	99
				
Knowledge	Pearson	-.240^*^	-.050	1
	Sig. (2-tailed)	.016*	.623	
	N	99	99	99

* . Correlation is significant at the 0.05 level (2-tailed).

## DISCUSSION

Our study shows that medical students' knowledge of autopsy is good and often begins with textbook descriptions and lectures, but first-hand experience is pivotal. Furthermore, the exposure to autopsy procedures provides students with first-hand insight into the intricate details of various diseases and their impact on the human body. This practical knowledge of autopsy procedures, objectives, and legal and ethical considerations is crucial for the medical curriculum to ensure competent healthcare professionals. These results align with a study by Taufan^9^ involving 76 medical students, which found that most respondents had good knowledge about malpractice and medicolegal forensics. All students participating in our study believed that the main objective of an autopsy is to establish the cause of death and their use as evidence in a court of law. However, it is unexpected that the students are still least comprehended about the special autopsy technique, the role of autopsies in confirming the deceased's identity, and the need for an inquest report for a medicolegal autopsy. In Malaysia, medicolegal or forensic autopsies do not require consent from the next of kin. This contrasts with clinical autopsies, where such consent is mandatory. Benbow^10^ stated that it is unacceptable that some medical students and doctors know little about autopsies and their uses. Medical students should know the appropriate facts about autopsies.

Attitudes towards autopsy can vary widely among medical students. Some may see it as a rite of passage and an essential learning experience. In contrast, others may approach it with apprehension due to cultural sensitivity or the emotional impact of handling deceased individuals. Educational programs must address these concerns and support constructive learning environments. There is limited and contradictory data about medical students' views towards autopsies.^11^ Medical students' perceptions of the emotional impact of the autopsy, its role in medical education, and the knowledge gained from attendance have been investigated using quantitative questionnaire surveys.^11,12^ Notably, many students positively perceive autopsy attendance as beneficial in medical education, exceptionally learning about the fallibility of medicine and merging clinical medicine with pathology and basic sciences.^12^ The detrimental emotional distress of autopsy attendance could be ameliorated with a compassionate introductory session that gives students an overview of the autopsy and the mortuary setting.^13^

There are also practical concerns that make using autopsies for teaching challenging. Firstly, hospital autopsies have declined dramatically over the last 50 years.^3^ Secondly, in recent years, changes in the philosophy of medical education delivery have put significant time pressure on the curriculum in most medical schools. In particular, there have been changes in the delivery and time allocation to specialties, especially forensic medicine and pathology.^14,15^ Some respondents commented on the lack of curriculum time for autopsy teaching and that scheduling autopsy sessions was challenging due to the lack of advanced notice of available autopsies. This explained why eight students never attended extra autopsy sessions during their two weeks of forensic posting rotation. The facilities at the mortuary for autopsies may also provide a technical challenge in delivering a health and safety-compliant environment where suitable numbers of students can be accommodated to maintain their engagement with the procedure.

For this reason, only a small percentage of students got the opportunity to assist the doctor during the autopsy. Participating in autopsies gives medical students a unique opportunity to comprehend the correlation between clinical symptoms and pathological findings. The findings of this study could have significant implications for medical education in Malaysia, shedding light on the effectiveness of current teaching methods and autopsy facilities. By understanding students' barriers and concerns regarding autopsy attendance, educational institutions can tailor their programs to address these issues and provide an environment that encourages active participation and learning.

Clinicopathological conference (CPC) involves presenting a patient's case, detailing both history and current medical records, along with pertinent data from laboratory tests, including biopsies results, treatments, and in cases that result in death, autopsy findings. This presentation is aimed at trainees and all physicians participating in the patient's care. Despite a decreasing rate of autopsies, the CPC remains a prevalent educational practice in numerous teaching hospitals. It serves as an ongoing education and teaching tool for medical students and physicians.^16^

Our teaching pedagogy in both universities assigned medical students to prepare a detailed presentation on the autopsy cases they have witnessed to foster active participation. This presentation should include a comprehensive presentation that consists of the case history, findings from the autopsy, and any relevant laboratory results. In addition, seminar classes are also conducted to encourage discussion on the insights gained from the autopsy and contribute to a deeper understanding of pathology and anatomy. The outcome of the seminars is to emphasize critical thinking and the ability to convey complex information in an accessible manner to both the students and their lecturer.

This study also found that Pearson’s statistical test revealed a significant correlation between the knowledge and students' attitudes toward autopsy. Students have positive feedback about forensic medicine postings because expectations, needs, motivation, and emotions influence them. Students' cultural backgrounds and beliefs can significantly affect their attitudes towards autopsies. Understanding how different cultures view death, the human body, and medical procedures is crucial in bridging gaps in acceptance and understanding of death. Acknowledging these cultural influences and gaining knowledge about diverse perspectives can help students appreciate the importance of autopsies in medical education, leading to greater acceptance and collaboration across varied cultural beliefs.

Based on this study, mandatory autopsy teaching should be integrated into the medical curriculum by embedding it as a core component of forensic medicine courses. Such integration could involve structured rotations in the forensic department, in our case, two weeks of forensic medicine posting, where students must attend a certain number of autopsies throughout their studies. Regular seminars could be held where students present and discuss these cases with their peers and lecturers, emphasizing the importance of correlating clinical findings with autopsy results. Assessment of this component is included in the student's grades to ensure they give it the necessary attention. Additionally, incorporating discussions about autopsies’ ethical and legal aspects would prepare students comprehensively for their future roles as physicians.

## CONCLUSIONS

Medical schools should strategically integrate autopsy attendance into their curricula to encourage constructive attitudes and practices. Preparatory sessions can be conducted to set expectations and address concerns, and reflections can be encouraged post-attendance to help students process their experiences and reinforce learning outcomes. This research seeks to provide valuable insights that can inform educational strategies and institutional support to enhance medical students’ participation and engagement in forensic teaching, ultimately contributing to their holistic medical education and professional development.

## References

[1] The Royal College of Pathologists (2002). Guidelines on autopsy practice. The Report of a Working Group of the Royal College of Pathologists..

[2] Razak NA, Haque M (2023). The Vital Role of Forensic Medicine as a ‘Hidden Curriculum’ in Medical Education: current Perspectives. Advances in Human Biology.

[3] Vanezis P (2021). Education and training in forensic medicine in United Kingdom: fit for purpose?. Med Sci Law.

[4] Loughrey MB, McCluggage WG, Toner PG (2000). The declining autopsy rate and clinicians’ attitudes. Ulster Med J.

[5] O’Grady G (2003). Death of the teaching autopsy. BMJ.

[6] Schmeling A, Kellinghaus M, Becker JC, Schulz R, Schäfer A, Pfeiffer H (2011). A web-based e-learning programme for training external post-mortem examination in curricular medical education. Int J Legal Med.

[7] O’grady G (2004). The Breakfast Club: case study of a teaching-autopsy curriculum. Med Teach.

[8] Burton JL, Scheimberg I, Bates AW, Lee A, Holbrook M, Morgan G (2001). Getting consent for necropsies: perhaps we should seek consent to show necropsies to students. BMJ.

[9] Taufan A. (2019). Hubungan tingkat pengetahuan dokter muda tentang jenis malpraktik dengan kurikulum hukum kesehatan pada stase forensik medikolegal. Jurnal Soshum Insentif.

[10] Bendow EW (1990). Medical students’ views on necropsies. J Clin Pathol.

[11] Anders S, Fischer-Bruegge D, Fabian M, Raupach T, Petersen-Ewert C, Harendza S (2011). Teaching post-mortem external examination in undergraduate medical education: the formal and the informal curriculum. Forensic Sci Int.

[12] Sergentanis TN, Papadodima SA, Evaggelakos CI, Mytilinaios DG, Goutas ND, Spiliopoulou CA (2010). Students’ physical and psychological reactions to forensic dissection: are there risk factors?. Anat Sci Educ.

[13] Bamber AR, Quince TA, Barclay SI, Clark JD, Siklos PW, Wood DF (2014). Medical student attitudes to the autopsy and its utility in medical education: A brief qualitative study at one UK medical school. Anat Sci Educ.

[14] Marshall R, Cartwright N, Mattick K (2004). Teaching and learning pathology: a critical review of the English literature. Med Educ.

[15] Johnson EO, Charchanti AV, Troupis TG (2012). Modernization of an anatomy class: from conceptualization to implementation. A case for integrated multimodal–multidisciplinary teaching. Anat Sci Educ.

[16] Zampieri F, Rizzo S, Thiene G, Basso C (2015). The clinico-pathological conference, based upon Giovanni Battista Morgagni’s legacy, remains of fundamental importance even in the era of the vanishing autopsy. Virchows Arch.

